# Integrated Network Pharmacology and Clinical Study to Reveal the Effects and Mechanisms of Bushen Huoxue Huatan Decoction on Polycystic Ovary Syndrome

**DOI:** 10.1155/2022/2635375

**Published:** 2022-05-13

**Authors:** Jie Ding, Mei Shanshan, Cai Mengcheng, Zhang Danying, Yu Jin

**Affiliations:** ^1^Department of Gynecology of Traditional Chinese Medicine, Changhai Hospital, Naval Medical University, Shanghai 200433, China; ^2^Traditional Chinese Medicine University of Shanghai, Shanghai 201203, China; ^3^International Peace Maternity and Child Health Hospital, School of Medicine, Shanghai Jiao Tong University, Shanghai 200025, China

## Abstract

**Objective:**

Bushen Huoxue Huatan Decoction (BHHD) is a classic prescription for treating polycystic ovary syndrome (PCOS). This study aims to explore the effects and possible mechanisms of BHHD on PCOS by integrating network pharmacology and clinical study.

**Methods:**

The components and potential drug targets of BHHD were analysed using the TCMSP platform, and the potential pathogenesis targets for PCOS were analysed using the GeneCards and OMIM databases. Subsequently, a disease-compound-target network diagram was established to identify the targets of BHHD treatment on PCOS. In addition, protein-protein interaction analysis, KEGG pathway analysis, and Gene Ontology biological analysis were carried out to reveal the mechanisms of BHHD. To further validate the analysis, a clinical trial involving 62 PCOS patients was conducted. All patients were treated with BHHD for 3 months and the ovulation rate, anthropometric indicators, clinical symptoms, and serological indicators were measured and compared before and after treatment.

**Results:**

The network pharmacology analysis showed that quercetin, luteolin, and kaempferol are the most significant active components in BHHD; STAT3, Jun, AKT1, MAPK3, MAPK1, and TP53 are the most critical drug targets; regulating hormones, reversing insulin (INS) resistance, exerting anti-inflammatory effects, and improving fertility might be the most important mechanisms of BHHD in the treatment of PCOS. After BHHD administration, the cyclic ovulation rate and the clinical symptoms including acanthosis nigricans and acne of patients were obviously improved. The serum endocrine levels of luteinising hormone (LH), LH/follicle-stimulating hormone, testosterone, dehydroepiandrosterone sulphate, insulin (INS), and area under the INS curve were evidently reversed, and the serum inflammatory factors levels including human interleukin (IL)-18, IL-16, IL-1*β*, IL-8, macrophage migration inhibitory factor, and human leukocyte differentiation antigen CD40 ligand were greatly reduced.

**Conclusion:**

BHHD has a good therapeutic effect on PCOS, and its mechanisms may be related to regulating hormone levels, improving insulin resistance, alleviating inflammation, and promoting pregnancy.

## 1. Introduction

Polycystic ovary syndrome (PCOS) is the one of the most prevalent endocrine and metabolic disorders in premenopausal women; it is characterised by hyperandrogenism and chronic anovulation [1]. PCOS affects about 6%–20% of women of reproductive age, and its symptoms often appear in early adolescence, with irregular menstrual cycles, acne, obesity, infertility, and so on. [2]. At present, the treatment of PCOS mainly includes surgery, long-term oral contraceptive, and ovulation induction; however, these treatments are limited by their high invasiveness, high recurrence rates, poor long-term results, and many side effects [3]. Recently, complementary and alternative medicine has become a more valuable option for the treatment of PCOS [4]. Traditional Chinese medicine (TCM) has a history of thousands of years in China and is favoured by both doctors and patients for its stable efficacy, good safety, and few side effects. Bushen Huoxue Huatan Decoction (BHHD) is an empirical prescription for PCOS in the Chinese medicine, which consists of 8 herbs including Paeoniae Radix and *Curcuma aromatica*. Good clinical effects have been achieved in the application of BHHD in long-term clinical practice; however, its mechanism remains unclear.

**Figure 1:**
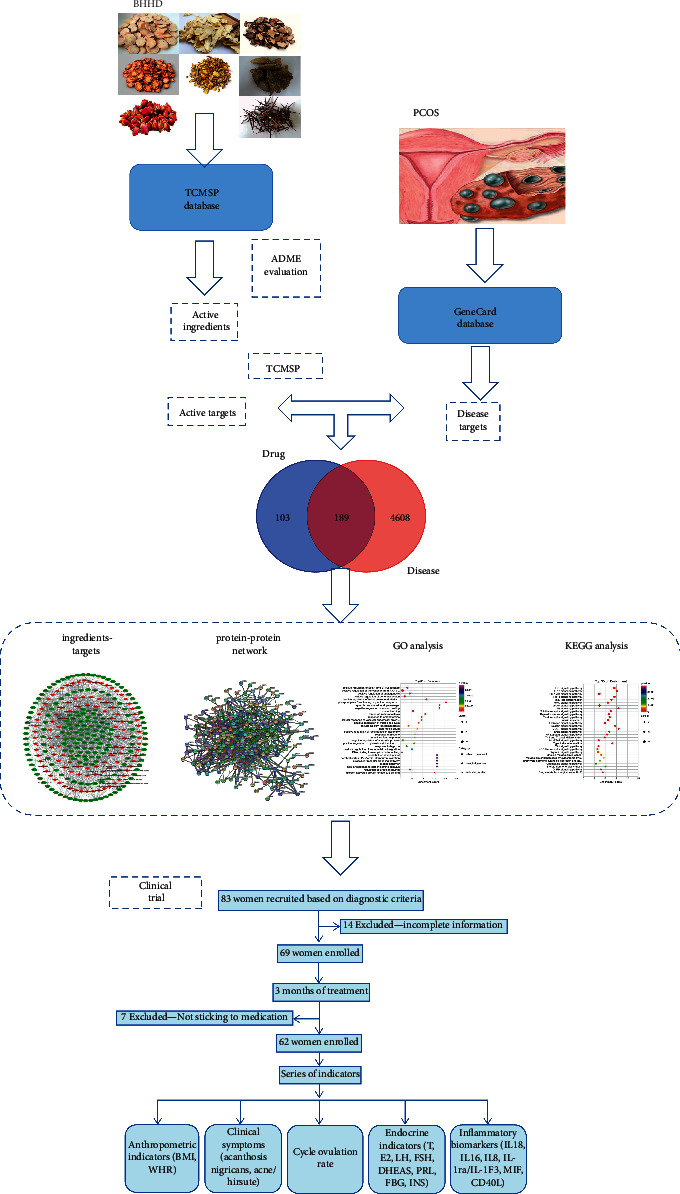
The idea and process of this research.

Network pharmacology, based on systems biology, multidirectional pharmacology, computational biology, network analysis, and other disciplines, fully combines pharmacology and informatics to explore the overall correlation between drugs and diseases, discover drug targets, and guide the development of new drugs [5]. Network pharmacology has the characteristics of integrity and systematicness, which is consistent with the holistic view and syndrome differentiation of TCM [6]. In recent years, network pharmacology has been widely used to explore the pharmacological mechanisms of TCM. In this study, the natural compounds of BHHD and their targets were analysed by network pharmacology so as to reveal the possible mechanisms of BHHD in the treatment of PCOS; besides, a clinical trial was conducted to further validate these analytical results. The experimental scheme design is shown in Figure 1.

## 2. Materials and Methods

### 2.1. Screening of Active Chemical Components

The Chinese herbal medicines contained in BHHD were searched using the TCMSP platform (http://lsp.nwu.edu.cn/tcmsp.php, 2021-4-7), a database containing the chemical compositions of Chinese medicines [7]. Oral bioavailability (OB) ≥ 30% and drug-likeness (DL) ≥ 0.18 were used as screening conditions for the active ingredients according to the requirements of the TCMSP database usage guidelines [8].

### 2.2. Target Screening of Potential Active Ingredients

To further analyse the molecular mechanism of BHHD for the treatment of PCOS, the TCMSP platform was used to obtain the target information of the potential active ingredients contained in BHHD. The full name of the gene was abbreviated by UniProt (https://www.uniprot.org/, 2021-4).

### 2.3. Analysis of Targets Related to PCOS Pathogenesis

The GeneCards database (https://www.genecards.org/, 2021-4-7) and OMIM database (https://omim.org/, 2021-4-7) were searched for genes related to PCOS using “polycystic ovary syndrome,” “Stein–Leventhal Syndrome,” and “PCOS” as the search terms [9, 10]. The results returned from both databases were merged and de-duplicated to obtain genes highly correlated with PCOS and unified in abbreviation format [11].

### 2.4. Construction of Component-Target-Disease Networks

Using the online Venn diagram tool (https://bioinfogp.cnb.csic.es/tools/venny/,2021-4), the intersection of disease targets and component targets was taken to obtain potential therapeutic targets, which were imported into Cytoscape software (version 3.7.1) to construct the component-target-disease correlation network.

### 2.5. Establishment of Protein-Protein Interaction (PPI) Network

To find the core targets in the network, the intersection was imported into STRING (https://cn.string-db.org/) to form the interaction relationship between target proteins. The PPI network was constructed with Cytoscape. The core targets were selected by calculating the degree value using Cytoscape's own analysis and drawn.

### 2.6. Gene Ontology (GO) and KEGG Enrichment Analysis

The GO database (http://geneontology.org/, 2021-4) was used to analyse significantly enriched biological process, molecule function, and cellular composition (*P* < 0.05). The signalling pathway of the target was analysed using the KEGG database (https://www.kegg.jp/, 2021-4) to identify significantly enriched pathways (*P* < 0.05). Visual analysis of GO and KEGG results was performed to discover the specific mechanism of BHHD in PCOS treatment.

### 2.7. Clinical Studies

#### 2.7.1. Clinical Patient Recruitment

PCOS patients were recruited from the outpatient clinic of the First Affiliated Hospital of Naval Military Medical University. The present study was approved by the ethics committee of this hospital and all enrolled patients met the inclusion and exclusion criteria, volunteered to participate in the clinical trial, and signed an informed consent.

#### 2.7.2. Diagnostic Criteria

The diagnostic criteria for PCOS are based on ESHRE/ASRM criteria [12]: (1) rare ovulation or anovulation, (2) clinical or biochemical hyperandrogenaemia, and (3) polycystic ovary morphology (defined as 12 or more [2–9 mm] follicles in each ovary identified via ultrasonography). Patients with compliance of at least two of the above diagnostic criteria after excluding the patients with congenital adrenal hyperplasia, diabetes, prolactinaemia, acromegaly, Cushing's syndrome, androgen-secreting neoplasm, and so on.

#### 2.7.3. Inclusion Criteria

Patients were included if they satisfied the (1) ESHRE/ASRM criteria; (2) did not use medications or stopped using medications for PCOS for more than 3 months and did not undergo diet or exercise therapy; and (3) agreed to voluntarily participate in the study and signed the informed consent form.

#### 2.7.4. Exclusion Criteria (Inclusion Process)

Patients were excluded if they had a (1) combination of serious primary diseases of the heart, liver, kidney, and haematopoietic system, as well as psychiatric disorders; (2) allergy and hypersensitivity to drugs contained in BHHD; and (3) incomplete information or inability to judge efficacy, affecting efficacy or safety evaluation, and so on.

#### 2.7.5. Exclusion Criteria (Experimental Procedure)

Patients were excluded if they (1) did not take the drug as prescribed or used it intermittently and (2) took the drug concomitantly with other drugs, which may interfere with the efficacy evaluation.

#### 2.7.6. Discharge Criteria

Patients who did not complete the clinical protocol as scheduled, including (1) those who withdrew on their own, (2) those who have been taking the drug for less than three menstrual cycles, and (3) those who have been removed by the investigator (e.g., patients with poor compliance or symptoms that make them unfit for continued treatment) were removed from the study.

#### 2.7.7. Treatment Method

Bushen Huoxue Huatan prescription is composed of Paeoniae Radix Alba (“Bai Shao” in Chinese, BS), *Curcuma aromatica* (“Yu Jin” in Chinese, YJ), Radix Salviae Miltiorrhizae (“Dan Shen” in Chinese, DS), Angelicae Sinensis Radix (“Dang Gui” in Chinese, DG), Scutellaria Baicalensis (“Huang Qin” in Chinese, HQ), Rosae Rugosae Flos (“Mei Gui” in Chinese, MG), Acori Tatarinowii Rhizoma (“Shi Chang Pu” in Chinese, SCP), and Spina Gleditsiae (“Zao Jiao Ci” in Chinese, ZJC) and its water decoction (BHHD) was prepared by the Pharmacy Department of the First Affiliated Hospital of Naval Military Medical University. One patient received 300 mL BHHD (including BS 24 g, DS 30 g, DG 24 g, HQ 9 g, MG 12 g, SCP 15 g, YJ 12 g, and ZJC 12 g) orally, twice a day, namely, 150 mL after breakfast and dinner. All PCOS patients were administered for 3 months and followed up for 3 months after treatment.

#### 2.7.8. Observation Indicators and Test Methods

The cyclic ovulation rate, clinical symptoms, endocrine and inflammatory levels of patients were compared before and after treatment. The observation indexes and test methods are as follows:

1. *Cyclic Ovulation Rate (COR)*. Basal body temperature was recorded throughout the trial period including treatment and follow-up, and COR was calculated as % = number of ovulated cycles/(number of ovulated cycles + number of nonovulated cycles) × 100%.
2. *Anthropometric Indexes and Clinical Symptoms*. The height, weight, waist circumference, and hip circumference of patients were measured, and changes in the body mass index [BMI, BMI (kg/m^2^) = weight (kg)/height (m^2^)] and waist-hip ratio [WHR, WHR = waistline (cm)/hipline (cm)] were calculated. In addition, the clinical symptoms including acne (based on the Rosenfield scoring system), hirsutism (based on the Ferriman–Gallwey scoring system) and acanthosis nigricans (refers to the darkening, thickening, and roughness of the skin in the neck, armpits, groin, and other skin folds) were comprehensively evaluated by two or more professional clinicians.
3. *Endocrine and Metabolic Parameters.* The serum levels of luteinising hormone (LH), follicle-stimulating hormone (FSH), testosterone (T), estradiol (E2), dehydroepiandrosterone sulphate (DHEAS), and prolactin (PRL) were detected by chemiluminescence immunoassay, the serum level of fasting blood glucose (FBG) was detected by the hexokinase method, and the serum INS concentrations at 0 min, 30 min, 60 min, 90 min, 120 min, and 180 min were detected by electrochemiluminescence. In addition, the area under the INS curve (IAUC) calculated according to the formula [IAUC = 0.5 × (INS_0 min + INS_180 min) + 0.75 × INS_60 min + INS_30 min + INS_120 min], INS resistance index (HOMA-IR) calculated according to the formula [HOMA-IR = fasting INS (FINS, INS_0 min, *μ*U/L) × FBG (mmol/L)/22.5], and INS sensitivity index (ISI) calculated according to the formula [ISI = 1/(FBG × INS_0 min)].
4. *Serum Inflammatory Cytokines*. The levels of serum inflammatory cytokines of PCOS patients (another eight healthy women were recruited as controls) were tested by Proteome Profiler Human Cytokine Array Kit (Catalog no. ARY005B) and analysed by Shanghai Uninvest Biotechnology Company; the significant inflammatory factors were verified by ELISA assay (Beyotime Systems).

## 3. Results

### 3.1. Network Pharmacology Analysis

#### 3.1.1. Components of BHHD Identification

A total of 1033 ingredients were obtained from BHHD based on the TCMSP platform, including 85 BS, 202 DG, 125 DS, 143 HQ, 121 MG, 105 SCP, 30 ZJC, and 222 YJ. According to OB ≥ 30% and DL ≥ 0.18, 142 potential active ingredients were screened out. The same ingredients were then removed from different herbs, resulting in 129 potential active ingredients.

#### 3.1.2. Common Targets of BHHD and PCOS Screening

**Figure 2:**
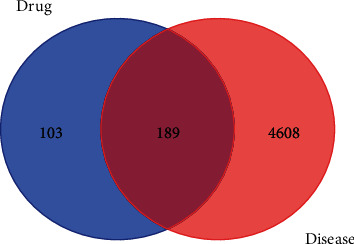
Venn diagram (blue is the drug targets; red is the disease targets; in the middle are common targets).

A total of 292 genes corresponding to potential active components of BHHD were screened using the targets information analysis function of TCMSP platform and 175 target genes were obtained after de-duplication. All these 175 target genes were annotated using UniProt data. In addition, a total of 4797 PCOS disease-related genes were gained from the GeneCards and OMIM database. Moreover, a total of 189 cross-targets genes for BHHD treatment of PCOS were captured through intersection analysis of drug components and PCOS disease-related genes.  Figure 2.

#### 3.1.3. Component-Target Network Analysis

**Figure 3:**
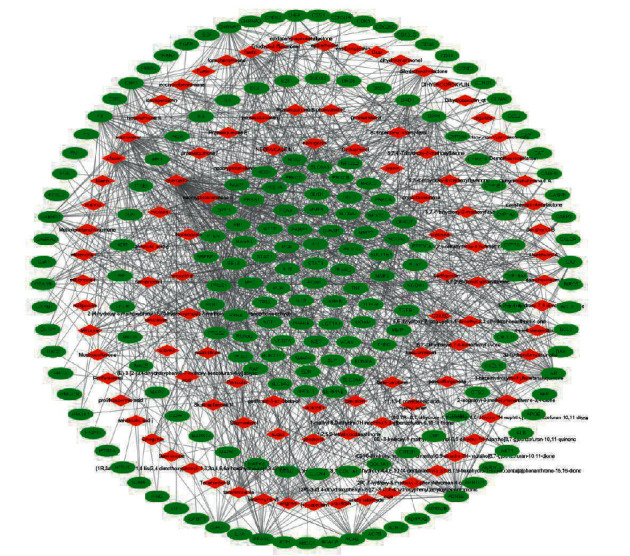
Component-target network (the red diamonds represent potential active ingredients, whereas the green circles represent target genes).

For further explanation, a component-target network was constructed, as shown in Figure 3. This network contains 106 potential active components of BHHD and 189 cross-targets. Nodes represent ingredients or targets, whereas edges represent the interactions between ingredients and targets. The top 10 ingredients are quercetin, luteolin, kaempferol, fisetin, wogonin, tanshinone IIA, naringenin, dihydrotanshinlactone, *β*-sitosterol, and baicalein, as shown in Table 1.

#### 3.1.4. PPI Network Analysis

**Figure 4:**
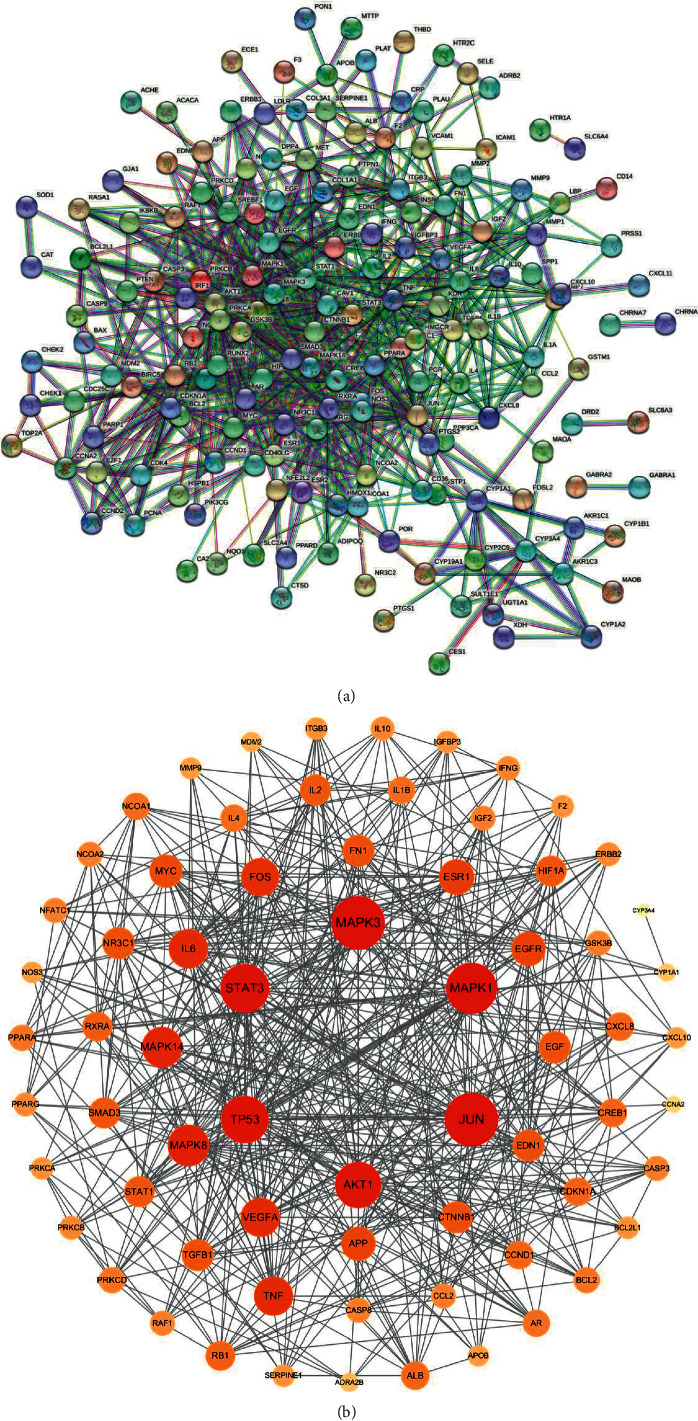
Results of core target screening. (a) PPI network diagram. (b) Core target diagram (the darker the node and the larger the area, the more important the target.).

It is important to accurately reveal and annotate all functional interactions between proteins in cells. We used PPI networks to explore the relationships between different target genes and to find key genes in the network. 189 cross-targets were imported into STRING and the species selected was *Homo sapiens* with a confidence level >0.900. There were 173 nodes and 1838 edges in Figure 4(a). The average node degree value was 10.62. The tsv format file of BHHD in PCOS treatment was exported from STRING and then imported into Cytoscape software for network topology parameter analysis. Nodes with degree values greater than 20 were selected as the key nodes for visualisation (Figure 4(b)). Target site-specific information is shown in Table 2.

#### 3.1.5. GO Functional Enrichment Analysis

**Figure 5:**
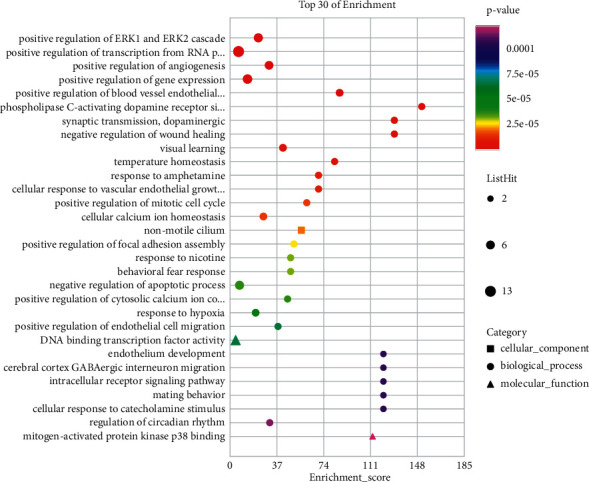
Bubble chart of enrichment analysis results. GO enrichment (top 30 of enrichment).

To elucidate the specific biological mechanisms of BHHD in PCOS treatment, 189 cross-targets for GO biological processes, including biological processes, molecular functions, and cellular components, were analysed using the GO database. The top 30 GO terms were selected for analysis based on the *P* value (Figure 5). The top 10 GO terms are positive regulation of ERK1 and ERK2 cascade (GO:0070374); positive regulation of transcription from RNA polymerase II promoter (GO:0045944); positive regulation of angiogenesis (GO:0045766); positive regulation of gene expression (GO:0010628); positive regulation of blood vessel endothelial cell migration (GO:0043536); phospholipase C-activating dopamine receptor signalling pathway (GO:0060158); synaptic transmission, dopaminergic (GO:0001963); negative regulation of wound healing (GO:0061045); visual learning (GO:0008542); and temperature homeostasis (GO:0001659).

#### 3.1.6. KEGG Pathway Analysis

**Figure 6:**
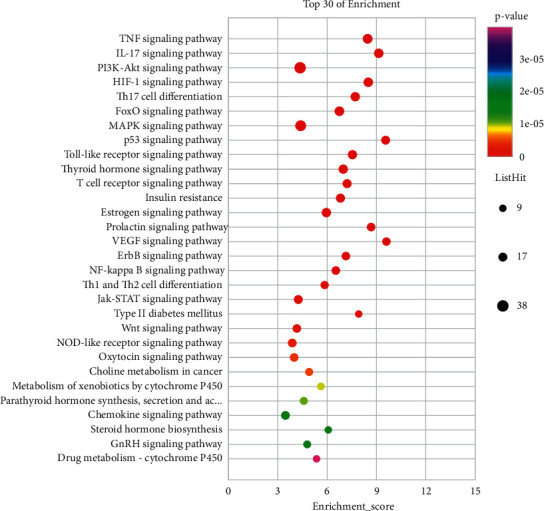
Bubble chart of enrichment analysis results. KEGG enrichment (top 30 of enrichment).

A total of 130 pathways were revealed by KEGG enrichment analysis with statistically significant differences (*P* < 0.05). Amongst them, several KEGG pathways were closely related to PCOS, including TNF signalling pathway (hsa04668), HIF-1 signalling pathway (hsa04066), FoxO signalling pathway (hsa04068), PI3K-Akt signalling pathway (hsa04151), Toll-like receptor signalling pathway (hsa04620), T-cell receptor signalling pathway (hsa04660), VEGF signalling pathway (hsa04370), p53 signalling pathway (hsa04115), INS resistance (hsa04931), ErbB signalling pathway (hsa04012), and oestrogen signalling pathway (hsa04915). The 30 pathways with the highest significance associated with PCOS are shown in Figure 6. The analysis of these pathways showed that BHHD exerts therapeutic effects on PCOS mainly through four aspects: anti-inflammatory immunity, improvement of INS resistance, regulation of hormone levels, and improvement of ovarian function.

### 3.2. Clinical Study

Based on the inclusion and exclusion criteria, 62 PCOS were actually included in this study. The mean age of patients was (26.21 ± 4.67) years, and the mean course of disease was (4.89 ± 5.02) years. This study was approved by the Ethics Review Committee of Chinese Registered Clinical Trials (CHIECRCT-20160050).

#### 3.2.1. Cycle Ovulation Rate

Basal body temperature was recorded throughout the trial period including treatment and follow-up (3 months after treatment). According to the calculation results, the ovulation rate of patients with PCOS after BHHD treatment was 61.2%, which was significantly increased compared with that before treatment (Table 3).

#### 3.2.2. Anthropometric Indexes and Clinical Symptoms

Our research showed that the prevalence rates of overweight (BMI >24 kg/m^2^), central obesity (WHR >0.8), hirsutism (F-G scoring >9), acne (Rosenfield scoring >0), and acanthosis nigricans (positive) were 28.5%, 61.3%, 45.2%, 77.4%, and 38.7%, respectively. After BHHD treatment, the clinical symptoms of acne and acanthosis nigricans were obviously improved (*P* < 0.05); however, there were no significant differences in BMI, WHR, and hirsutism before and after treatment (*P* > 0.05) (Tables 4 and 5).

#### 3.2.3. Endocrine and Metabolic Parameters

Our test results showed that the serum levels of LH, LH/FSH, T, DHEAS, INS_30min_, INS_90min_, INS_120min_, INS_180min_, and IUAC were significantly reduced after BHHD treatment (*P* < 0.05) (Tables 6–8), whereas the serum levels of FSH, E_2_, PRL, FBG, INS_0min_, INS_60min_, HOMA-IR, and ISI were no obvious difference before and after treatment.

#### 3.2.4. Serum Inflammatory Cytokines

The protein microarray detection results showed that the expression levels of CCL5/RANTES, CD40 ligand/TNFSF5, complement component C5/C5a, CXCL1/GRO*α*, CXCL12/SDF-1, IL-1ra/IL-1F3, IL-8, IL-13, IL-16, IL-18/IL-1F4, MIF, and serpin E1/PAI-1 were obviously increased in PCOS compared with the healthy women (*P* < 0.05) (Table 9). As ELISA analysis results showed that, the serum levels of IL-18, IL-16, IL-1*β*, IL-8, MIF, and CD40 were significantly reduced after BHHD treatment (*P* < 0.05) (Table 10).

## 4. Discussions

PCOS is the most common endocrine and metabolic disorder that disturbs the physical and reproductive health of women of childbearing age [13]. Therefore, the exploration of effective treatment and drugs for PCOS is one of most important research hotspots in the field of gynaecology and endocrinology. In recent years, the rise of complementary and alternative medicine, especially TCM, has enriched the development of PCOS therapeutics. BHHD is a classic and empirical prescription for the treatment of PCOS developed according to the theory of TCM. Our previous studies found that BHHD could downregulate serum testosterone levels in androgen-sterilised rats by upregulating the expression of the androgen metabolising enzyme P450arom [14]; BHHD could improve INS sensitivity and INS resistance by increasing the expression of INS receptor substrate-1 and tyrosine phosphorylation in adipose tissue of rats [15]. These studies preliminarily explored the possible mechanisms of BHHD in the treatment of PCOS; however, the components of BHHD are complex and these studies are far from sufficient. Therefore, it is imperative to explain the mechanisms of BHHD systematically and completely. Nowadays, with the development of systems biology, multidirectional pharmacology, computational biology, and other technologies, network pharmacology emerged at the right moment, and in view of its integrity and systematic characteristics, network pharmacology has been widely used in the pharmacology research of TCM. In this study, network pharmacology techniques were used to explore the specific mechanisms of BHHD in the treatment of PCOS. It is worth noting that, to verify the analysis results of network pharmacology, a clinical trial was conducted to further clarify the mechanisms of BHHD.

### 4.1. The Core Components of BHHD

According to the “compound-target-disease” network diagram, the core components of BHHD were obtained in our study, and the top 10 ingredients were quercetin, luteolin, kaempferol, fisetin, wogonin, tanshinone IIA, naringenin, dihydrotanshinlactone, *β*-sitosterol, and baicalein. Quercetin is a flavonol compound with multiple biological activities and is widely distributed in the plant kingdom. Pourteymour et al. reported [16] that Quercetin could treat PCOS by reducing serum T and LH levels in patients with antioxidant and anti-inflammatory effects; Wang et al. [17] found that Quercetin could alleviate chronic inflammation in PCOS model rats by mimicking the effect of estrogen, reducing INS level and improving the INS resistance. Luteolin is one of the most biologically active flavonoids widely found in nature, with anti-inflammatory, antitumor, antibacterial, antiviral, and other pharmacological effects. Huang and Zhang [18] shown that luteolin could improve INS resistance of PCOS by promoting the PI3K/AKT signalling pathway, and besides, luteolin could alleviate oxidative stress of PCOS by restoring the Nrf2 pathway to enhance antioxidant response.

Baicalein, a flavonoid glycoside isolated from *S. baicalensis*, has shown biological activities against several androgen-associated disorders, such as prostate cancer, androgenetic, and acne. Interestingly, our previous research revealed that baicalin would potentially be an effective therapeutic agent for hyperandrogenism in PCOS by inhibiting the recruitment of GATA1 to the HSD3B2 promoter in ovarian tissue [19]. In addition, other ingredients, such as kaempferol, wogonin, and tanshinone IIA, have anti-inflammatory, antioxidant, or androgen effects to varying degrees. In summary, these studies lay a solid foundation for the mechanism exploration of BHHD in the treatment of PCOS.

### 4.2. BHHD Benefited Pregnancy in PCOS Patients

It is known that about two-thirds of PCOS patients ovulate sparsely or even anovulate, which is the main cause of infertility in PCOS. In recent years, assisted reproductive technology has greatly improved the pregnancy rate of PCOS; however, there are several problems, such as high incidence of ovarian hyperstimulation syndrome, low endometrial receptivity, and increased risk of abortion [20], so it makes sense to find ways to be safer [21, 22]. Our network pharmacology analysis suggested that BHHD is involved in progesterone-mediated ovum maturation, VEGF signalling pathways, and several hormonal pathways, suggesting its potential in improving fertility. The core components of BHHD have participated in the development of follicular and the regulation of endometrial receptivity. For instance, previous studies showed that Quercetin could maintain the morphological integrity of developing follicles by improving the antioxidant capacity of the ovary, and besides, it could minimise the apoptosis of granulosa cells, maintain the ability of oocytes, and promote follicular maturation [23, 24]. What is more, Quercetin could remove senile decidual cells and enhance the decidual ability of embryonic stem cell, thereby reducing endometrial receptivity disorder and infertility caused by embryo implantation [25]. Kaempferol could maintain follicular survival through the PI3K pathway, increase the level of active mitochondria, and promote meiosis recovery of oocytes, thereby stimulating follicular development [26]. The benefits of BHHD for pregnancy in PCOS patients were confirmed in our clinical trial, where cycle ovulation rates in PCOS patients increased significantly after BHHD treatment. The findings suggest that BHHD can increase a woman's chances of conceiving without obvious side effects.

### 4.3. BHHD Restored the Disrupted Hormones in PCOS

Hormone disorder is an important feature of PCOS. Gonadotropin-releasing hormone, androgen, estrogen, growth hormone, cortisol, parathyroid hormone, calcitonin, and other hormones are out of balance in PCOS patients [27]. Androgen overactivation is not only a clinical feature, but also a risk factor of PCOS [28]. For example, PCOS patients with overactivation are mainly manifested as ovulation disorder, menstrual disorder, hirsutism, and acne. Oestrogen inhibits ovulation, and high levels of oestrogen can cause damage to the lipi-STAT3 pathway, resulting in failed implantation of PCOS mouse embryos and hindering conception [29]. LH is a gonadotropin that stimulates follicle cell proliferation and produces large amounts of androgens that are not fully converted to oestrogen, further increasing the production of extraglandular aromatised estrone. Excess androgens lead to follicular atresia, fibrosis, and thickening of the ovarian envelope, which can impair ovulation [30]. Impaired ovarian steroid synthesis can also lead to an excess of androgens. Patients with PCOS were found to be deficient in 3*β*-hydroxysteroid dehydrogenase, 17-steroid reductase, and aromatase P450. The lack of these enzymes leads to impaired ovarian steroid synthesis, failure of ovarian androgen conversion to oestrogen, and increased androgen levels, which prevent ovulation [31]. Multiple hormones interact to cause the occurrence or development of PCOS.

Our network pharmacology analysis results showed that estrogen signalling pathway, GnRH signalling pathway, ovarian steroid production pathway, and other hormone-related signalling pathways were significantly expressed, suggesting that BHHD may have a certain regulatory effect on various hormones. The effects of natural compounds in regulating hormones have always been a research hotspot. Quercetin can inhibit the proliferation and apoptosis of bovine ovarian cells and the release of IGF-I, progesterone, and testosterone; reverse the effect of xylene on ovarian function; and protect ovarian function [32]. Kaempferol shows androgen-like activity and acts as a selective androgen receptor modulator to antagonise androgen effects [33]. Kaempferol is also a natural progesterone receptor modulator that activates progesterone receptor signalling in vitro and in vivo [34]. Luteolin and its derivatives can inhibit oestrogen biosynthesis by reducing aromatase expression and destroying stable aromatase protein, but they do not affect the catalytic activity of aromatase. Luteolin and its derivatives are natural oestrogen inhibitors [35, 36]. These natural compounds have different effects on hormone regulation in different diseases, and further research is needed to elucidate their functions [37]. Our clinical study found a significant decrease of DHEAS and T levels in PCOS after BHHD treatment. Consistently, the clinical symptoms of PCOS caused by androgen overactivation, acne and acanthosis nigricans, also improved obviously. Besides, the serum levels of LH and LH/FSH were significantly reduced after treatment, suggesting that the gonadal axis function of patients has been restored to a certain extent.

### 4.4. BHHD Reversed Insulin Resistance in PCOS

Insulin resistance (IR) and compensatory hyperinsulinemia are also important endocrine features in PCOS patients, with an incidence of about 70%. INS regulates the activity of enzymes in the ovaries and liver and its signals are interconnected through a variety of intracellular pathways; at present, IRSS, PI3K/AKT, and MAPK are generally considered to be key nodes of INS function. These nodes are also active in the ovary and their interaction with FSH and LH signalling pathways allows INS to directly regulate ovarian function [38, 39]. Previous reports showed that IR is involved in the excessive production of androgens, causing low-grade inflammation [40]. Obesity and IR aggravate hyperandrogenemia in patients, creating a vicious cycle that promotes the development of PCOS [41]. Both the PI3K/AKT pathway and MAPK pathway were marked as significant pathways in our network pharmacology analysis, which suggested that BHHD could directly regulate INS-related pathways, such as FoxO signalling pathway, INS resistance, type II diabetes, and INS signalling pathway. FoxO1 regulates the effects of INS on glucose metabolism and influences other hormone signalling pathways and has a broad role in some aspects of lipid metabolism [42]. The key components of IR and hyperinsulinemia are well studied. Quercetin interacts with many molecular targets in the small intestine, pancreas, skeletal muscle, adipose tissue, and liver to control systemic glucose homeostasis by inhibiting glucose uptake and INS secretion, increasing INS sensitivity and improving glucose utilisation in peripheral tissues [43]. luteolin improves IR by normalising pancreatic islet function and regulating plasma glucagon-like peptide-1 and gastric inhibitory polypeptide levels [44]. Kaempferol is a natural antidiabetic compound that increases the activity of Akt and hexokinase in the liver and decreases the activity of pyruvate carboxylase and glucose-6 phosphatase, thereby inhibiting glucose production and improving INS sensitivity [45]. In our clinical study, the improvement of INS sensitivity related parameters confirmed the improvement of BHHD on INS-related pathways. In the INS release test, INS levels decreased over multiple time periods, indicating increased INS utilisation and improved IR in patients after BHHD intervention.

### 4.5. Anti-Inflammatory and Immune Effects of BHHD

Recent studies show that chronic low-grade inflammation is closely associated with PCOS. Inflammatory processes are involved in ovulation and play a key role in follicular dynamics. Visceral adipose tissue induces inflammatory responses and maintains the inflammatory state of adipose cells by increasing the recruitment of inflammatory cytokines, monocyte chemotactic proteins, and immune cells. The TNF signalling pathway and NOD-like signalling pathway are classical inflammation-related pathways. TNF-*α* levels are higher in PCOS patients and are directly related to body fat percentage levels [46]. The activation of the TNF-*α* system triggers ovarian apoptosis and reduces oocyte capacity, affecting follicle maturation and elimination, thereby reducing fertility in patients with PCOS [44]. At the same time, increased TNF-*α* can directly reduce the capacity of endometrial tissue glucose uptake, affect endometrial function, and lead to infertility [47]. In terms of NOD-like signalling pathways, high levels of androgens stimulate chronic low-grade inflammation in the ovary, activating NLRP3 inflammatory vesicles and inducing ovarian granulosa scorching, follicular dysfunction, and ovarian interstitial cell fibrosis. This is accompanied by the secretion of large amounts of IL-1*β* and IL-18, further enhancing the inflammatory environment [48].

Our protein chip analysis showed that compared with healthy women, PCOS patients had an inflammatory state with significantly increased serum levels of inflammatory cytokines CCL5/RANTES, CD40 ligand/TNFSF5, complement component C5/C5a, CXCL1/GRO*α*, CXCL12/SDF-1, IL-1ra/IL-1F3, IL-8, IL-13, IL-16, IL-18/IL-1F4, MIF, and serpin E1/PAI-1. These findings confirm the presence of chronic inflammation in PCOS patients. While, after BHHD intervention, the serum levels of inflammatory cytokines IL-1ra/IL-1F3, IL-8, IL-16, IL-18, MIF, and CD40L in PCOS patients were significantly reduced. According to previous studies, IL-1ra/IL-1F3 is an endogenous inhibitor of IL-1; it can bind to IL-1RI and IL-1RII, but it does not initiate signal transduction and thus functions as a competitive inhibitor of the activity of IL-1 through IL-1R. A large number of animal studies have shown that IL-1ra can reduce the occurrence of low blood sugar levels generated by colony-stimulating factor due to endotoxemia, as well as strengthen organic tolerance [49]. IL-8, a proinflammatory chemokine secreted by microglia, astrocytes, and endothelial cells, is regarded as one of the most studied chemokines that plays a significant role in the development of atherosclerosis and future cardiovascular diseases [50], and elevated IL-8 is thought to be associated with IR in PCOS [51]. IL-16 is a proinflammatory cytokine secreted by macrophages, CD8 T cells, and epithelial cells. IL-16, a chemoattractant, leads to the homing of other immune cells at the site of injury and inflammation, resulting in an oxidative burst which subsequently causes oxidative stress [52]. Il-18 is a proinflammatory cytokine that belongs to the IL-1 superfamily and is closely associated with insulin resistance, metabolic syndrome, and is an important predictor of long-term cardiovascular mortality. Rudnicka et al. reported that the concentration of IL-18 is increased in PCOS patients regardless of presence of insulin resistance and obesity; however, obese women with hyperinsulinemia have even higher concentrations of IL-18 [53]. MIF is the first cytokine identified as a pleiotropic inflammatory mediator and is regulated by multiple immune cells, including macrophages, B cells, and T cells [54]. Elevated MIF activates the NF-*κ*B pathway in the ovary and promotes the expression of IL-1*β*, IL-6, iNOS, and TNF-*α* [55]. CD40 is expressed in a variety of cells including B cells, thymic epithelial cells, and macrophages and interacts with CD40L in a variety of immune responses [56]. Serum CD40L levels are significantly higher in patients with PCOS than in normal women and are positively correlated with C-reactive protein and IR levels [57]. The core components of BHHD are flavonoids with outstanding anti-inflammatory capacity. Quercetin inhibits TNF-*α*-induced apoptosis and inflammation by blocking the nuclear factor-*κ*B (NF-*κ*B) and AP-1 signalling pathways. Luteolin inhibits proinflammatory mediators such as IL-1*β*, IL-6, IL-8, IL-17, IL-22, TNF-*α*, and COX-2. It also regulates various signalling pathways, such as NF-*κ*B, JAK-STAT, and TLR [58]. All of these studies contribute to BHHD's anti-inflammatory immunity.

## 5. Conclusion

This study is the first to identify the potential of BHHD in the treatment of PCOS from a network pharmacology perspective and reveal the mechanism of BHHD action in four aspects, namely, regulating hormone levels, reversing insulin resistance, anti-inflammatory immunity, and promoting pregnancy in patients. It is noteworthy that these aspects have been confirmed in our subsequent clinical trial. In addition, this study also found that monomer compounds such as quercetin and kaempferol have excellent performance in all aspects of PCOS treatment and may become new hot drugs for clinical treatment of PCOS if more rigorous pharmacological and clinical studies are carried out.

## Figures and Tables

**Table 1:** Compound information sheet.

Molecule ID	Molecule name	Structure	OB (%)	DL	Degree	Herb
MOL000098	Quercetin	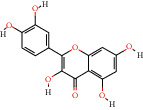	46.43	0.28	113	MG, ZJC
MOL000006	Luteolin	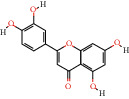	36.16	0.25	44	DS
MOL000422	Kaempferol	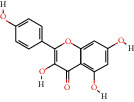	41.88	0.24	44	BS, ZJC, SCP
MOL013179	Fisetin	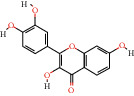	52.6	0.24	35	ZJC
MOL000173	Wogonin	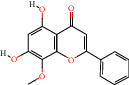	30.68	0.23	33	HQ
MOL007154	Tanshinone IIa	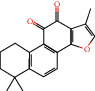	49.89	0.4	27	DS
MOL004328	Naringenin	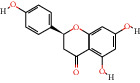	59.29	0.21	26	YJ
MOL007100	Dihydrotanshinlactone	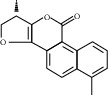	38.68	0.32	24	DS
MOL000358	*β*-Sitosterol	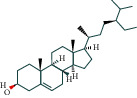	36.91	0.75	24	ZJC, BS, DG, HQ, MGH, YJ
MOL002714	Baicalein	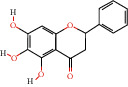	33.52	0.21	21	HQ

**Table 2:** Information on 20 core targets.

Target	Description	UniProt ID	Degree	Status
STAT3	Signal transducer and activator of transcription 3	P40763	45	Reviewed
JUN	Jun proto-oncogene, AP-1 transcription factor subunit	P05412	43	Reviewed
AKT1	AKT serine/threonine kinase 1	P31749	42	Reviewed
MAPK3	Mitogen-activated protein kinase 3	P27361	42	Reviewed
MAPK1	Mitogen-activated protein kinase 1	P28482	40	Reviewed
TP53	Tumour protein P53	P04637	39	Reviewed
TNF	Tumour necrosis factor	P01375	33	Reviewed
MAPK8	Mitogen-activated protein kinase 8	P45983	31	Reviewed
MAPK14	Mitogen-activated protein kinase 14	Q16539	30	Reviewed
IL-6	Interleukin-6	P05231	29	Reviewed
EGFR	Epidermal growth factor receptor	P00533	28	Reviewed
FOS	Fos proto-oncogene, AP-1 transcription factor subunit	P01100	28	Reviewed
APP	Amyloid beta precursor protein	P05067	27	Reviewed
VEGFA	Vascular endothelial growth factor A	P15692	27	Reviewed
ESR1	Oestrogen receptor 1	P03372	26	Reviewed
CTNNB1	Catenin beta 1	P35222	24	Reviewed
EGF	Epidermal growth factor	P01133	24	Reviewed
CXCL8	C-X-C motif chemokine ligand 8	P10145	22	Reviewed
EDN1	Endothelin 1	P05305	22	Reviewed
MYC	MYC proto-oncogene, BHLH transcription factor	P01106	21	Reviewed

**Table 3:** Comparison of ovulation in patients with PCOS before and after treatment (%).

	Cases	Total cycles (number)	Ovulated cycles (number)	Nonovulated cycles (number)	Ovulation rate (%)
Before treatment	62	372	107	265	28.8
After treatment	62	372	227	144	61.2^*∗*^

*Note.* ^*∗*^ *P* < 0.05.

**Table 4:** Comparison of anthropometric indexes ($\bar{X}$ ± *S*).

	Cases	BMI	WHR
Before treatment	62	22.034 ± 3.64	0.84 ± 0.07
After treatment	62	21.44 ± 3.06	0.83 ± 0.07

**Table 5:** Comparison of clinical symptoms (%).

	Cases	Hirsutism	Acne	Acanthosis nigricans
Before treatment	62	28 (45.2)	48 (77.4)	24 (38.7)
After treatment	62	26 (41.9)	36 (58.1)^*∗*^	11 (17.7)^*∗*^

*Note.* ^*∗*^Continuous variables were presented as mean ± SD. Satterthwaite was applied when the *P*-value for *F*-test (equality of variance test) was <0.05. A *P*-value <0.05 was considered to be statistically significant in two-tailed test.

**Table 6:** Serum sex hormone changes before and after treatment ($\bar{X}$ ± *S*).

	Cases	LH (IU/L)	FSH (IU/L)	LH/FSH	T (*μ*g/L)	E2 (pg/mL)	DHEAS (*μ*g/dL)	PRL (*μ*g/L)
Before treatment	62	9.65 ± 5.77	6.31 ± 1.70	1.58 ± 0.92	0.58 ± 0.28	46.73 ± 29.10	294.21 ± 94.75	12.13 ± 6.48
After treatment	62	7.63 ± 5.63^*∗*^	6.79 ± 1.20	1.11 ± 0.70^*∗*^	0.50 ± 0.13^*∗*^	42.00 ± 19.81	250.20 ± 71.86^*∗*^	11.16 ± 5.04

*Note.* ^*∗*^Continuous variables were presented as mean ± SD. Satterthwaite was applied when the *P*-value for *F*-test (equality of variance test) was <0.05. A *P*-value < 0.05 was considered to be statistically significant in two-tailed test.

**Table 7:** Serum sex hormone changes before and after treatment ($\bar{X}$ ± *S*).

	Cases	INS0 min	INS30 min	INS60 min	INS90 min	INS120 min	INS180 min
Before treatment	62	10.81 ± 7.07	76.23 ± 44.19	78.57 ± 48.59	77.04 ± 51.12	64.18 ± 40.30	27.68 ± 22.78
After treatment	62	10.40 ± 5.07	50.69 ± 35.30^*∗*^	65.54 ± 38.09	60.55 ± 24.71^*∗*^	45.23 ± 26.32^*∗*^	20.89 ± 12.50^*∗*^

*Note.* ^*∗*^Continuous variables were presented as mean ± SD. Satterthwaite was applied when the *P* value for *F* test (equality of variance test) was <0.05. A *P*-value <0.05 was considered to be statistically significant in two-tailed test.

**Table 8:** Comparison of INS before and after treatment ($\bar{X}$ ± *S*).

	Cases	FBG (mmol/L)	HOMA-IR	IAUC	ISI
Before treatment	62	5.04 ± 0.46	2.61 ± 2.17	217.56 ± 106.96	0.0276 ± 0.196
After treatment	62	5.00 ± 0.45	2.35 ± 1.27	160.73 ± 81.13^*∗*^	0.0281 ± 0.194

*Note.* ^*∗*^Continuous variables were presented as mean ± SD. Satterthwaite was applied when the *P*-value for *F*-test (equality of variance test) was <0.05. A *P*-value < 0.05 was considered to be statistically significant in two-tailed test.

**Table 9:** Inflammatory cytokines with significantly different INT values between PCOS and healthy women detected by proteome profiler human cytokine array kit ($\bar{X}$ ± *S*).

Index	Density INT (mm^2^)		*P*-value
	PCOS	Health women	
Reference spots	20778.8 ± 1593.6	21674.2 ± 2436.9	0.1961
CCL5/RANTES	22124.6 ± 1277.8	25117.3 ± 2715.2	0.0005^*∗*^
CD40 ligand/TNFSF5	17456.6 ± 3125.6	14630.6 ± 4630.7	0.0159^*∗*^
Complement component C5/C5a	14752.9 ± 6610	8783.3 ± 9464.3	0.0143^*∗*^
CXCL1/GRO*α*	7409.8 ± 5930.9	0.0 ± 0.0	0.0000^*∗*^
CXCL12/SDF-1	13251.6 ± 4410.3	10547.8 ± 3355.7	0.0036^*∗*^
IL-1ra/IL-1F3	10950.8 ± 2491.8	0.0 ± 0.0	0.0000^*∗*^
IL-8	12428.8 ± 23671.1	19921.4 ± 33480	0.0374^*∗*^
IL-13	1763.2 ± 2766.3	6372.6 ± 4389.7	0.0044^*∗*^
IL-16	2579.3 ± 3031.3	6000.3 ± 5001.8	0.0203^*∗*^
IL-18/IL-1F4	6950.9 ± 4118.1	16923.0 ± 1831.2	0.0000^*∗*^
MIF	18353.9 ± 2793.1	16376.1 ± 3268.7	0.0340^*∗*^
Serpin E1/PAI-1	18189.9 ± 7078.6	22070.2 ± 1389.7	0.0051^*∗*^

*Note.* ^*∗*^Continuous variables were presented as mean ± SD. Satterthwaite was applied when the *P*-value for *F*-test (equality of variance test) was <0.05. A *P*-value <0.05 was considered to be statistically significant in two-tailed test.

**Table 10:** Comparison of inflammatory factors before and after treatment ($\bar{X}$ ± *S*).

	PCOS cases	IL-18 (pg/mL)	IL-16 (pg/mL)	IL-8 (pg/mL)	IL-1ra/IL-1F3 (pg/mL)	MIF (pg/mL)	CD40 L (pg/mL)
Before treatment	62	97.22 ± 13.25	102.41 ± 8.97	103.61 ± 13.54	133.42 ± 13.93	66.73 ± 9.18	65.48 ± 5.84	
After treatment	62	53.95 ± 10.96^*∗*^	54.34 ± 9.03^*∗*^	50.78 ± 11.30^*∗*^	61.94 ± 16.38^*∗*^	27.73 ± 6.12^*∗*^	31.80 ± 6.28^*∗*^	

*Note.* ^*∗*^Continuous variables were presented as mean ± SD. Satterthwaite was applied when the *P*-value for *F*-test (equality of variance test) was <0.05. A *P*-value <0.05 was considered to be statistically significant in two-tailed test.

## Data Availability

The data used to support the findings of this study are included within the article.

## Abbreviations

BHHD: Bushen Huoxue Huatan decoction
PCOS: Polycystic ovary syndrome
TCMSP: Traditional Chinese Medicine Systems Pharmacology Database and Analysis Platform
KEGG: Kyoto Encyclopedia of Genes and Genomes
GO: Gene ontology
INS: Insulin
LH: Luteinising hormone
FSH: Follicle-stimulating hormone
T: Testosterone
DHEAS: Dehydroepiandrosterone sulphate
SD: Standard deviation
INT: Intensity
BMI: Body mass index
CD40 ligand/TNFSF5: Human leukocyte differentiation antigen CD40 ligand
CXCL1/GRO*α*: Melanoma growth-stimulating factor/growth-regulating oncogene *α*
CXCL2/SDF‐1: C‐X‐C motif chemokine ligand 12/stromal cell-derived factor-1
IL-1r*α*/IL-1F3: Human interleukin 1 receptor *α*
IL-8: Human interleukin 8
IL-13: Human interleukin 13
IL-6: Human interleukin 16
IL-18/IL-1F4: Human interleukin 18
MIF: Macrophage migration inhibitory factor
PAI-1: Plasminogen activator inhibitor 1.
